# Supplementary material for: Hybrid Offspring of C57BL/6J Mice Exhibit Improved Properties for Neurobehavioral Research
Source: eNeuro. 2022 Aug 17;9(4):ENEURO.0221-22.2022. doi: 10.1523/ENEURO.0221-22.2022 (PMC9395247; doi:10.1523/ENEURO.0221-22.2022)
Supplement: Extended Data Figure 1-2 — Results of phenotyping assays for every age group and strain. aValues indicate median [IQR] for every age group, strain, and metric. For learning on the MWM, values indicate mean ± SEM. Download Figure 1-2, DOCX file. [file enu-eN-NWR-0221-22-s07.docx]

**Extended data tables**

**Extended Data Figure 1-2**

**Extended Data for Figure 1. Results of phenotyping** **assays for every age group and strain**

| **Phenotype** | **Test** | **Metric** | **C57, 3M**^a^ | **FVB, 3M**^a^ | **HYB, 3M**^a^ | **C57, 9M**^a^ | **FVB, 9M**^a^ | **HYB, 9M**^a^ |
| --- | --- | --- | --- | --- | --- | --- | --- | --- |
| Size | - | Weight [g] | 27  [25 28] | 31  [30 32] | 32  [30 35] | 30  [30 32] | 37  [34 38] | 45  [41 47] |
| **Sensorimotor behavior** | | | | | | | | |
| Visual behavior | Optomotor drum | Number of head turns | 7  [3 11] | 0  [0 0] | 7  [5 10] | 8  [5 11] | 0  [0 0] | 8  [5 9] |
| Motor endurance | Treadmill | Distance to failure [cm] | 227  [148 242] | 120  [73 197] | 222  [30 262] | 91  [49 120] | 40  [29 64] | 126  [59 164] |
| Balance | Rotarod | Latency to fall [s] | 260  [195 278] | 235  [194 246] | 140  [107 147] | 165  [141 186] | 122  [89 123] | 188  [139 221] |
| **Psychiatric-like behavior** | | | | | | | | |
| Anxiety-like behavior | Elevated plus maze | Contra-anxiety index | -0.59  [-0.66 -0.55] | -0.44  [-0.55 0.38] | -0.34  [-0.55 0.22] | -0.56  [-0.75 0.46] | -0.28  [-0.43 0.05] | -0.14  [-0.27 0.15] |
| Depressive-like behavior | Forced swim test | Freeze duration [s] | 124  [94 135] | 209  [130 262] | 201  [85 290] | 129  [100 167] | 0  [0 14] | 138  [95 188] |
| Exploration | Open field | Activity [%] | 0.23  [0.17 0.27] | 0.19  [0.15 0.22] | 0.28  [0.2 0.31] | 0.35  [0.33 0.38] | 0.3  [0.27 0.33] | 0.35  [0.28 0.37] |
| **Cognitive behavior** | | | | | | | | |
| Learning | MWM | Latency to platform [s] | 21.7±4.3 | 41.1±3.8 | 19.3±3.9 | 33.3±2.9 | 42.1±4 | 17.8±2.9 |
|  |  | Distance traveled [m] | 9.3±2.6 | 14.7±1.9 | 11.1±3.2 | 12.7±1.2 | 11.2±0.6 | 16.7±4.6 |
| Spatial long-term memory | MWM, probe day | Fraction of time in the target quadrant | 0.29  [0.23 0.36] | 0.21  [0.09 0.28] | 0.29  [0.19 0.39] | 0.3  [0.24 0.4] | 0.25  [0.15 0.31] | 0.55  [0.36 0.62] |
|  |  | Fraction of visits to the target quadrant | 0.28  [0.26 0.27] | 0.21  [0.15 0.3] | 0.36  [0.33 0.44] | 0.34  [0.27 0.4] | 0.28  [0.25 0.36] | 0.45  [0.36 0.5] |

^a^Values indicate median [IQR] for every age group, strain, and metric. For learning on the MWM, values indicate mean±SEM.

**Extended Data Figure 5-1**

**Extended Data for Figure 5. Mice used for electrophysiological recordings**

| **Animal** | **Sex** | **Strain** | **Driver line**^a^ | **Reporter line**^a^ | **Viral vector** | **Age**^b^ **[week]** | **Weight**^b^ **[g]** | **Probe** | **Neocortex sessions** | **CA1 sessions** |
| --- | --- | --- | --- | --- | --- | --- | --- | --- | --- | --- |
| mP23 | M | C57 | PV-Cre | Ai32 | - | 15 | 31.2 | Buzsaki32 | - | 14 |
| mDS1 | M | C57 | CaMKII-Cre | Ai32 | - | 14 | 25.7 | Dual-sided64 | - | 5 |
| mDS2 | F | C57 | CaMKII-Cre | Ai32 | - | 30 | 24.2 | Dual-sided64 | - | 21 |
| mC41 | M | HYB | CaMKII-Cre | Ai32 | - | 10 | 33.7 | Stark64 | 6 | 33 |
| mA234 | M | HYB | CaMKII-Cre | Ai32 | - | 16 | 30 | Buzsaki32 | 15 | 24 |
| mB142 | M | HYB | PV-Cre | - | DIO-ChR2 | 21 | 35.9 | Buzsaki32 | 1 | - |

^a^For C57 animals, the male parent was the reporter, and the female parent was the driver.

^b^At time of implantation.

**Extended Data Figure 5-2**

**Extended Data for Figure 5. Linear track behavior and place coding in individual mice**

| **Animal** | **Strain** | **Sessions** | **Trials**^a^ | **Speed [cm/s]** | **Active PYR**^b^ | **SI**^c^  **[bits/s]** | **Place cells** | **Field size [cm]** | **Phase precessing fields** | **Precession slope size [cyc/cm]** | **Precession effect size** |
| --- | --- | --- | --- | --- | --- | --- | --- | --- | --- | --- | --- |
| mC41 | HYB | 33 | 195  [159 245] | 45  [33 56] | 532 | 1.8  [0.7 3.7] | 317/532 (60%) | 40  [22 57] | 207/393  (53%) | 0.02  [0.01 0.03] | 1.5  [1.2 2] |
| mA234 | HYB | 24 | 171  [135 206] | 29  [21 33] | 428 | 0.8  [0.4 2.3] | 191/428 (45%) | 42.5  [32 57] | 146/256  (57%) | 0.02  [0.02 0.03] | 1.6  [1.3 2.1] |
| **Summary** | **HYB** | **57** | **181**  **[145 222]** | **34**  **[27 49]** | **960** | **1.3**  **[0.5 3.3]** | **508/960 (53%)** | **40**  **[25 57]** | **353/649**  **(54%)** | **0.02**  **[0.02 0.03]** | **1.5**  **[1.2 2.1]** |
| mP23 | C57 | 14 | 117  [99 140] | 23  [18 31] | 80 | 1  [0.4 1.9] | 32/80 (40%) | 40  [31 57] | 25/43  (58%) | 0.02  [0.02 0.02] | 1.5  [1.3 2.4] |
| mDS1 | C57 | 5 | 136  [120 140] | 23  [22 25] | 44 | 0.5  [0.2 0.9] | 10/44 (23%) | 56.2  [31 64] | 9/16  (56%) | 0.02  [0.01 0.02] | 1.4  [1.2 3.1] |
| mDS2 | C57 | 19 | 161  [130 182] | 23  [18 29] | 655 | 1.5  [0.7 3.1] | 401/655 (61%) | 40  [27 55] | 274/544  (50%) | 0.02  [0.01 0.03] | 1.6  [1.3 2.1] |
| **Summary** | **C57** | **36** | **135**  **[108 174]** | **23**  **[19 29]** | **779** | **1.4**  **[0.6 2.9]** | **443/779 (57%)** | **40**  **[27 57]** | **308/603**  **(51%)** | **0.02**  **[0.01 0.03]** | **1.6**  **[1.3 2.1]** |

^a^Number of one direction trials, median [IQR] over sessions.

^b^Well-isolated PYR, active and stable on the linear track.

^c^SI, spatial information.

**Extended Data Figure 6-2**

**Extended Data for Figure 6. Optogenetic activation of individual neurons in individual mice**

| **Animal** | **Region** | **Sessions** | **Activated PYR^a^** | **Locally activated PYR^b^** | **Activated INT** | **Locally activated INT** |
| --- | --- | --- | --- | --- | --- | --- |
| mA234 | CA1 | 24 | 493/1807 (27%) | 280/577 (48%) | 122/284 (43%) | 34/76 (45%) |
| mC41 | CA1 | 31 | 720/3761 (19%) | 442/950 (46%) | 119/782 (15%) | 58/197 (30%) |
| **Total** |  | **55** | **1213/5568 (22%)** | **722/1527 (47%)** | **241/1066 (23%)** | **92/273 (34%)** |
| mA234 | Neocortex | 15 | 653/1203 (54%) | 235/327 (72%) | 208/456 (46%) | 60/120 (50%) |
| mC41 | Neocortex | 6 | 82/343 (24%) | 39/82 (48%) | 21/61 (34%) | 6/11 (55%) |
| **Total** |  | **21** | **735/1564 (47%)** | **274/409 (67%)** | **229/517 (44%)** | **66/131 (50%)** |

^a^Well-isolated PYR with increased firing rate during optogenetic stimulation (p<0.05, Poisson test), out of all PYR recorded in the same sessions.

^b^Light-activated PYR recorded on the illuminated shank.
